# The Impact on Anxiety Symptoms of an Immersive Virtual Reality Remediation Program in Bipolar Disorders: A Randomized Clinical Trial

**DOI:** 10.3390/jcm13144203

**Published:** 2024-07-18

**Authors:** Diego Primavera, Antonio Urban, Elisa Cantone, Marcello Nonnis, Cesar Ivan Aviles Gonzalez, Alessandra Perra, Massimo Tusconi, Federica Sancassiani

**Affiliations:** 1 Department of Medical Sciences and Public Health, University of Cagliari, Monserrato Blocco I (CA), 09042 Cagliari, Italyalessandra.perra@unica.it (A.P.);; 2 University Hospital of Cagliari, 09042 Cagliari, Italy; 3 Department of Pedagogy, Psychology, Philosophy, University of Cagliari, 09123 Cagliari, Italy; marcello.nonnis@unica.it; 4 Department of Nursing, Universidad Popular del Cesar, Valledupar 200001, Colombia

**Keywords:** bipolar spectrum, bipolar disorder, anxiety, virtual reality, remediation, advanced technologies laboratory

## Abstract

**Background**: The objective of this work is to investigate the effectiveness of a cognitive remediation intervention on anxiety symptoms in people with bipolar disorder and the therapeutic effect on people whose anxiety symptoms were above the threshold for a screener and whose comorbidity could be identified as an anxiety disorder. **Methods**: The experimental intervention included 24 sessions (around 45 min each), two for each week over three months. The entire program was inspired by user-centered rehabilitation principles in a recovery-oriented perspective and an approach to bipolar disorder in an evolutionary and non-discriminating vision. The primary outcomes measure the score of the Zung Self-Rating Anxiety Scale (SAS), hypothesizing a higher decrease in the experimental group than in the control group. The survey has been conducted per the CONSORT guidelines for feasibility studies. **Results**: We evaluate a decrease in the overall SAS score from T0 to T1 to be higher in the experimental group compared to the control group, indicating an improvement in anxiety symptoms (*p* < 0.0001). **Conclusions**: The study suggests that virtual reality could have a role in treating anxiety symptoms and disorders in young adults with bipolar disorders or anxiety symptoms in people with hyperactivity and novelty-seeking behaviorsunder stress and high risk for bipolar disorder.

## 1. Introduction

Bipolar spectrum disorders show a frequency of around 2–4% in community surveys [1,2,3]. These disorders must be considered a relevant public health issue due to their impact on individuals, families, and society and their consequences on the work, social network, and social functioning of the people who suffer from them [2]. The course of bipolar spectrum disorders is characterized by acute episodes of mania, depression, or mixed symptoms followed by periods (even long) with subthreshold mood symptoms frequently associated with some anxiety symptoms [4]. Bipolar spectrum and, to a greater extent, bipolar disorder type 2 (characterized by episodes of hypomania and depression) can persist undiagnosed and untreated or erroneously treated for years [5,6]. Several factors can contribute to delaying the diagnosis of bipolar spectrum disorder. Firstly, the stigma causes unacceptance of awareness of suffering from the disorders; another factor is that the onset often occurs with a depressive episode [7], inducing an erroneous diagnosis of major depressive disorder and inappropriate treatment [8,9]. Unfortunately, antidepressants, the first choice for the treatment of major depressive disorder, have less efficacy and can aggravate the course of bipolar disorders, with a high risk of inducing a switch from depression to mania [9,10]. Indeed, the occurrence of anxiety symptoms and disorders, especially in episodes of hypomania [11], can lead to accepting only a “reassuring” diagnosis of anxiety disorder. The diagnosis of anxiety disorders seems to allow the person with unrecognized bipolar disorder to consent to removal, in the sense of not being aware of the threat of the “other” diagnosis; one does not know or does not want to know the concept that there can frequently be comorbidity. The result is often still the taking of antidepressants (several antidepressants are the first choice for the treatment of anxiety disorders). The delay of the diagnosis occurs ten years on average, and a large number of people with this disorder have been mistreated [5,6].

Treatments for bipolar disorders with recognized efficacy and effectiveness are currently available [12]. However, the outcome of the disease is not always positive; in some cases, after a crisis, it has shown a return to premorbid levels, at a good level of wellness and functioning; in other cases, the evolution is towards a chronic functional impairment and a condition of marked vulnerability to new episodes of crisis [13,14]. Suffering with bipolar disorder is associated with a high risk of suicide [15] and an average decrease in life expectancy of 13 years [16].

One determinant of the unfavorable course and adverse outcomes of bipolar disorder is comorbidity with anxiety disorders [4,17]. Comorbidity with an anxiety disorder is associated with a greater frequency of critical episodes and recurrences [18] and an increased risk of suicide attempts [19,20]. This element is relevant when considering that more than half of people who have bipolar disorder have a comorbid anxiety disorder during their lifetime [21] and that anxiety disorders manifest themselves during the course, even in the phases of euthymia [4].

Comorbidity between bipolar disorder and anxiety spectrum disorders has been proposed in the literature [22,23,24], as encompassed within a normal course of illness [25]. This observation is supported by the relevant proportion of bipolar disorder patients who have a marked co-presence of symptoms attributable to anxiety [26]. Based on the National Comorbidity Survey, it emerges that 95.5 percent of people with bipolar I disorder have diagnostic criteria meeting three or more additional psychiatric disorders; these additional disorders include conduct disorders, substance use disorders, and, to a greater extent, anxiety symptoms [25,27,28].

In the context of a complex diagnosis such as bipolar disorder, the relevance of comorbid conditions as a risk factor emerges; in particular, anxiety in its various epiphenomena represents a significant impact factor in the trajectory of the course because of its negative impact, which encompasses a greater complexity of treatment [29,30,31].

A multiplicity of studies have produced data inherent to bipolar disorder, reporting high rates of overall comorbidity, with a marked focus on anxiety disorders, highlighting how multiple subtypes of anxiety disorder are found within this cluster [32].

The negative effects represented by anxiety disorders in comorbidity with bipolar disorder [33,34] are seen markedly in terms of an intensification of bipolar symptoms and any other disorders in further comorbidity, resulting in an overall worsening of the general condition [35,36]. There is evidence of a lowering of the age of onset of bipolar disorder, together with a reduced response to pharmacological treatment, an increased rate of suicide, an increase in substance use, and an overall worsening of quality of life [37]. The persistence of anxiety symptoms in the euthymic stages of bipolar disorder is an essential determinant of relapse [38]. The use of a virtual reality program is considered optimal in that it allows for the improvement of anxiety symptoms, limiting their effect on generating relapses [39]. Moreover, not being an antidepressant, it is not subject to the risk of switching in the expansive phase. Finally, not being a drug, the question of drug interactions with other treatments that may already be in place does not arise [40].

Anxiety disorders can be easily treated with antidepressants [41,42] and psychotherapy [42]. However, antidepressants are challenging to manage in the context of pharmacological therapy for bipolar disorder, and their use can lead to an increase in the dosage of mood stabilizers [43]. On the other hand, effective psychotherapeutic treatment for anxiety disorders is not always available, and the treatment of a person with bipolar disorder may require specific skills.

In this framework, it seemed helpful to examine in detail the results on anxiety symptoms (secondary outcome) of an immersive virtual reality protocol structured to counteract cognitive decline in people with bipolar disorder (primary outcome results were already published).

The objective of this work is to investigate the effectiveness in people with bipolar disorder of a cognitive remediation intervention on anxiety symptoms and the therapeutic effect on people whose anxiety symptoms were above the threshold for a screener and whose comorbidity could be identified as an anxiety disorder.

## 2. Materials and Methods

### 2.1. Design

This study is a secondary analysis focused on the results of anxiety symptoms and anxiety disorders of a randomized controlled feasibility trial [44,45] with a cross-over design, regularly registered (NCT05070065, ClinicalTrials.gov 2021, accessed on 30 May 2024). Because of the complex nature of the study, multiple professionals were involved, in addition to those included in the ClinicalTrials.gov registration, to leverage multiple skills and obtain more accurate results. The intervention carried out using the CEREBRUM 3.0.1 software was part of a more extensive mindfulness intervention where mindfulness-based cognitive therapy for bipolar disorder was leveraged to achieve effects on cognitive functioning [46]. The survey was conducted per the CONSORT guidelines for feasibility studies [47].

### 2.2. Sample

The people treated in the RCT had a clinical diagnosis of bipolar I or II disorder according to DSM-IV criteria [48]. They were randomly selected among the patients attending the “Centro di Psichiatria di Consultazione e Psicosomatica” of the University Hospital of Cagliari. People with ages between 18 and 75 years, of all sexes, were admitted. The choice of this age group was due to the marked difference in the clinical aspects of people under 18 years of age; in addition, for the age group above 75 years, this limit was chosen to avoid possible cognitive impairments that are more detectable with increasing age. The enrollment phase of the study began on 10 November 2021; the study ended on 10 February 2022, as reported on ClinicalTrials.gov. An informed consent form had to be signed before the intervention. People having a current manic or depressive episode and/or severe eye diseases or epilepsy were excluded, as the simulation by virtual reality could negatively impact these diseases. A biometrician blinded the names of the participants and carried out the randomization of the sample into two arms (for experimental and control groups) by a computerized program. He did not participate in the subsequent steps of the trial. The researchers carrying out the psychometric evaluation were blinded to the type of intervention the evaluated participant had undergone.

### 2.3. Experimental Intervention and Control Group

The “virtual reality” intervention consists of a fully immersive cognitive remediation path adapted for this population. The previously published papers have a detailed description of the software [44,45]. We chose to use CEREBRUM 3.0.1 software, a virtual reality program used in psychiatric rehabilitation conceived and designed by PRoMIND (Rome) in association with IDEGO-Virtual Psychology (Rome). Such Virtual Reality software is fully immersive and was produced by clinicians and experts specializing in cognitive rehabilitation. It is used through the Oculus Go virtual reality visor, a CE-marked device developed by Meta in collaboration with Qualcomm and Xiaomi. The Oculus Go looks like an all-in-one headset, containing all the components needed to provide virtual reality experiences, and does not need to be connected to an external device. In addition, the CEREBRUM 3.0.1 app allows users to immerse themselves in virtual scenarios that simulate everyday reality and home and urban scenarios [44,45]. The tasks proposed to participants were of increasing difficulty. The clinician can modulate the difficulty of the exercise, adapting to each participant’s performance and functional skills. This methodology was adopted to make the intervention more effective and stimulating and less frustrating.

The experimental intervention included 24 sessions (around 45 min each), two for each week over three months. The intervention was strictly manualized; the entire program was inspired by user-centered rehabilitation principles [49], in a recovery-oriented perspective [50,51], and an approach to bipolar disorder in an evolutionary and non-discriminating vision [52,53,54]. People with bipolar disorder may sometimes be noncompliant with complex rehabilitation programs. In contrast, this type of solution through virtual reality is simple to administer and engaging, leading to a high level of compliance with the proposed protocol [55,56]. A treatment, as usual, was delivered (psychiatric colloque and care, pharmacotherapy with or without psychotherapy) to each participant, both from the control group and from the experimental group. The experimental group received the virtual reality intervention plus treatment as usual and was compared with the control group, which received only treatment as usual. Then, the control group, as per the cross-over design, also received the experimental treatment.

### 2.4. Outcome and Study Tools

The primary outcome measures of this study (secondary in the deposited protocol) are: (1) the score on the Zung’s Self-Rating Anxiety Scale (SAS) [57] (the hypothesis was that there was a greater decrease in the experimental group than in the control group); (2) people with a score on the Zung’ Self-Rating Anxiety Scale above the cut-off identifying a “case” of anxiety disorder (the hypothesis was that there was a greater decrease in people with symptoms of anxiety of clinical relevance in the experimental group than in the control group).

The Zung Self-Rating Anxiety Scale is a self-administered tool consisting of 20 items covering both psychological anxiety features and somatic anxiety components. Responses are given in Likert 1–4-point rank, in which 1 is none, never, or for a small amount of time, and 4 is most of the time or all of the time. Participants were asked to indicate their answers over the last week. Items include negative and positive questions concerning experiences, with positives having a reverse score. Scoring ranged from 20 to 80. The SAS has good psychometric properties, including internal consistency with Cronbach’s alpha  =  0.82 [58]; concurrent validity with Pearson correlation of r  =  0.30 with the Taylor Manifest Anxiety Scale [57]; and accuracy in discriminating between clinical and non-clinical samples and anxiety and other psychiatric disorders (cut-off > 45) [57].

### 2.5. Statistical Analysis

The change in the SAS total score (in the overall sample experimental and control sample and subsamples of participants with comorbid chronic diseases) and of each SAS item within the group by time (T0 vs. T1) was calculated as a difference in the mean score ± standard deviation; the differences in score change of SAS total score and each SAS item T0 (before treatment) vs. T1 (after treatment) between groups were measured by ANOVA 1-way statistics. In this feasibility study context, because of the sample size, it was chosen to proceed with an ANOVA that allowed for the assessment of differences over time and between groups; in the later stages of the study, the sample would be increased, and multivariate analyses would be conducted. The nominal variables were compared using Fisher’s exact test, and chi-square with Yates correction was needed. 

The analysis of the change in frequency of participants over the SAS score detecting anxiety disorders in the groups from T0 to T1 was carried out using the method of analysis of variance for nominal data by Castellan [59]. A *p*-value < 0.05 was considered statistically significant.

## 3. Results

The analysis was finally carried out on 39 individuals of the experimental sample out of 50 people recruited (with 11 dropouts in the experimental group at follow-up) and 25 individuals in the control group (without people dropping out). The choice of such a balance of the study sample is due to its specific nature as a feasibility cross-over study. Statistical analyses were conducted as part of a cross-over study.

No statistically significant differences were found between groups by mean age, frequency of sex, and degree of education (see Table 1), both in total groups and in participants with comorbid chronic diseases; in the experimental group, seven suffered from chronic thyroid disease; three from neurological disorders; four from cardiovascular diseases; three from autoimmune or rheumatological diseases; three from female genital disorders; two from spinal hernias; two from chronic gastrointestinal diseases; and one from diabetes (some participants suffered from multiple disorders). In contrast, the control group comprised two individuals with chronic thyroid disease, one with cardiovascular disease, one with neurological disorders, two with autoimmune or rheumatological diseases, three with female genital disorders, one with diabetes, and two with chronic gastrointestinal diseases (some individuals experienced multiple disorders).

Table 2, Table 3 and Table 4 show a decrease in the overall SAS score from T0 to T1, higher in the experimental group compared to the control group (indicating an improvement in anxiety symptoms) (F = 61.984 *p* < 0.0001). 

However, the improvement is heterogeneous in the different items of the scale. A more significant drop in the score in the experimental group with statistically significant differences against the control group is noted only in 10 out of 20 items: 1 (I feel more nervous and anxious than usual) (F = 109.003 *p* < 0.0001), 2 (I feel afraid for no reason at all) (F = 7.770, *p* = 0.007), 3 (I get upset easily or feel panicky) (F = 29.607, *p* < 0.0001), 4 (I feel like I am falling apart and going to pieces) (F = 197.532, *p* < 0.0001), 7 (I am bothered by headaches neck and back pain) (F = 22.669, *p* < 0.0001), 8 (I feel weak and get tired easily) (F = 52.156, *p* < 0.0001), 12 (I have fainting spells or feel like it) (F = 9.095 *p* = 0.004), 13 (I can breathe in and out easily) (F = 22.630, *p* < 0.0001), 15 (I am bothered by stomach aches or indigestion) (F = 39.816, *p* < 0.0001), 16 (I have to empty my bladder often) (F = 10.132, *p* = 0.002), and 17 (My hands are usually dry and warm) (F = 51.309, *p* < 0.0001). On the contrary, in four items, the control group improves more than the experimental group in the specific anxiety symptom as in items: 10 (I can feel my heart beating fast) (F = 10.48.5 *p* = 0.002), 18 (My face gets hot and blushes) (F = 36.592, *p* < 0.0001), 19 (I fall asleep quickly and get a good night’s rest) (F = 24.262, *p* < 0.0001), 20 (I have nightmares) (F = 6.201, *p* = 0.015).

The difference in the improvement in anxiety from T0 to T1 disappears if we compare the two subgroups, experimental and control, in which there was comorbidity with another chronic non-psychiatric illness (F = 0.917, *p* = 0.342, Table 5) while, on the contrary, the improvement is substantial in participants without comorbid chronic non-psychiatric illnesses (F = 71.101, *p* < 0.0001, Table 5). This improvement is appreciable, taking into account the lack of participants without comorbid non-psychiatric illnesses of, for example, vascular disruptions that can lead to cognitive impairment [60].

The number of participants with anxiety symptoms below the threshold for anxiety disorder decreases after treatment in the experimental group (−17.9%) compared to the control group (0 decreasing) (Table 6, Fisher’s exact test, *p* = 0.025). Also, in the analysis of this outcome measure the differences disappear if we compare the two subgroups, experimental and control, in which there was comorbidity with another chronic non-psychiatric illness (Table 6, Fisher’s exact test, *p* = 0.111), on the contrary the improvement remains strong in participants without comorbid chronic non-psychiatric illnesses (Table 6, Fisher’s exact test *p* = 0.025).

## 4. Discussion

A cognitive remediation program conducted to counteract cognitive decline in people with bipolar disorder shows that it can unexpectedly lower the extent of anxiety symptoms associated with bipolar disorder and reduce these symptoms to the point that, compared to the control group, after the intervention, in the experimental group participants who would screen positive for comorbid anxiety disorder, i.e., with a threshold score on the SAS scale, showed a decrease. The results do not appear to be replicated in participants with chronic non-psychiatric illnesses. The anxiety symptoms that improve the most are those linked to the so-called psychological component (Items 1–6). While some psychosomatic anxiety items improve, others do not; they do not appear to improve sleep-related anxiety symptoms (Items 19 and 20), which worsen. This is an essential limitation because the dysregulation of the sleep–wake rhythm can represent an element of vulnerability towards relapses in bipolar disorders [61,62,63]. However, the secondary analysis relating to the effect of virtual reality on all personal and social rhythms (in this same trial) highlighted that the effect was still significant on overall rhythms, even if, consistent with the results of the present analysis on the efficacy of anxiety symptoms, a low specific effect on sleep rhythm was highlighted [64]. Indeed, the specific sleep rhythm in bipolar disorders is altered in a linear correlation with the duration of the disease [65], and is of relevance in the bipolar disorders of older adults and people with increasing risk of late-onset bipolar disorder [66,67]. These elements, together with the low impact in cases with comorbidity with chronic non-psychiatric illness, whose association with bipolar disorders increases with age [68,69], suggest that intervention with virtual reality must target, at least in a future experimental phase, young people or even conditions below the diagnostic threshold in vulnerable people [70,71]. Firstly, even more than at other stages of life, especially in adolescents and young adults, the use of antidepressants is even more contraindicated and was associated with an increased risk of suicide; in fact, in 2004, the United States Food and Drug Administration issued a black-box warning on antidepressants in adolescents and young adults, indicating that they were associated with an increased risk of suicide [72]. Despite numerous controversies, this caution warning in youth prescribing remains in force [73]. SSRI and SNRI antidepressants are the first choice for treating some anxiety disorders [41]. However, for young people, the choice of a non-pharmacological treatment should be a priority, if proven effective.

Regarding the limited improvement in specific anxiety symptoms, while the experimental group showed significant improvements in overall anxiety symptoms, specific items related to psychosomatic and sleep-related anxiety did not show significant improvements. This heterogeneity in the results indicates that the intervention may not be equally effective for all types of anxiety symptoms.

Furthermore, with regard to comorbidity considerations, the study shows that improvements in anxiety symptoms are less pronounced in participants with comorbid chronic non-psychiatric illnesses. This suggests that the intervention may need to be tailored or supplemented for individuals with multiple health conditions.

Some significant limitations that interfere with the possibility of generalizing the results are attributable to the small sample size. However, given the almost complete absence of literature data on this specific intervention in a specific population, the objective of the study was to perform a feasibility analysis and derive a preliminary measure of improvement that subsequent studies can confirm.

Thus, the results of this study suggest that virtual reality interventions could have a role in young adults with bipolar disorders or at risk for bipolar disorders, remembering that anxiety disorder comorbidity is associated with a higher frequency of recurrence in bipolar disorders [18] and with a higher risk of suicide [19,20]. Furthermore, not only were anxiety disorders found, even during the phases of euthymia of the bipolar course [4], but, especially in youth and young adults, anxiety disorders are often a precursor to the onset of bipolar disorder [74,75].

Future studies must, therefore, be aimed at studying the effect of virtual reality in the initial phases of bipolar disorder on anxiety and the repercussions on the course of the mood disorder, or even the effect in young adults with hyperactivity and novelty seeking [76,77,78] and under stress with anxiety symptoms that exhibit characteristics of high risk for bipolar disorder [79,80].

## 5. Conclusions

The study suggests that virtual reality could have a role in treating anxiety symptoms and disorders in young adults with bipolar disorders or in anxiety symptoms of people with hyperactivity and novelty seeking under stress and high risk for bipolar disorder. As highlighted in the literature, our results emphasize that an improvement in anxiety symptoms influences the overall course of bipolar disorder [81,82,83]. Future studies are needed to confirm the preliminary results of this secondary outcome study.

## Figures and Tables

**Table 1:** Study Sample.

	Experimental Arm	Control Arm	Statistics
Sex (Female)	14 (64.1%)	7 (72%)	Chi-square 1 df = 0.431 *p* = 0.512
Age Mean Years ± SD	47.51 ± 13.52	46.28 ± 13.40	F 1; 63 df = 0.127; *p* = 0.723
At least 13 years of education	24 [61.5%]	16 [64%]	Chi-square 1 df = 0.039 *p* = 0.843
Female participants with chronic diseases	15 (78.9%)	8 (80%)	Chi-square Yates Correction 1 df = 0.001 *p* = 0.999
Age of participants with chronic diseases	52.26 ± 12.89	49.8 ± 12.84	ANOVA 1way 1; 28 df F = 0.639, *p* = 0.239

**Table 2:** Differences in modifications in SAS Global Score and each SAS item score by time, comparing between groups—Part 01.

Experimental Group Control Group	T0	T1	Differences ANOVA 1-way 1; 63 df
T0 SAS Global (N = 39) Exp Group	61.05 ± 17.22	53.64 ± 17.61	−7.41 ± 3.33
T1 SAS Global (N = 25) Control Group	56.92 ± 15.69	57.96 ± 16.90	+1.04 ± 4.16
			F = 61.984, *p* < 0.0001
(N = 39) Exper SAS Item 1	2.72 ± 0.74	2.29 ± 0.86	−0.43 ± 0.13
(N = 25) Contr SAS Item 1	2.48 ± 1.02	2.60 ± 1.01	0.12 ± 0.20
I feel more nervous and anxious than usual.			F = 109.003, *p* < 0.0001
(N = 39) Exper SAS Item 2	2.12 ± 1.09	1.82 ± 1.12	−0.20 ± 0.16
(N = 25) Contr SAS Item 2	2.08 ± 1.05	2.00 ± 1.13	−0.08 ± 0.18
I feel afraid for no reason at all.			F = 7.770, *p* = 0.007
(N = 39) Exper SAS Item 3	2.23 ± 1.09	1.97 ± 1.07	−0.26 ± 0.17
(N = 25) Contr SAS Item 3	2.00 ± 1.16	2.16 ± 1.12	+0.16 ± 0.21
I get upset easily or feel panicky.			F = 29.607, *p* < 0.0001
(N = 39) Exper SAS Item 4	2.55 ± 1.04	2.05 ± 1.14	−0.50 ± 0.15
(N = 25) Contr SAS Item 4	2.04 ± 1.07	2.16 ± 1.12	+0.12 ± 0.15
I feel like I am falling apart and going to pieces.			F = 197.532, *p* < 0.0001
(N = 39) Exper SAS Item 5	3.26 ± 0.85	3.02 ± 0.94	−0.24 ± 0.16
(N = 25) Contr SAS Item 5	3.36 ± 0.74	3.20 0.80	−0.16 ± 0.15
I feel that everything is all right and nothing bad will happen.			F = 0.999, *p* = 0.321
(N = 39) Exper SAS Item 6	1.92 ± 0.99	1.84 ± 1.07	−0.08 ± 0.14
(N = 25) Contr SAS Item 6	1.88 ± 0.14	1.92 ± 1.01	+0.04 ± 0.13
My arms and legs shake and tremble.			F = 1.314, *p* = 0.356
(N = 39) Exper SAS Item 7	2.57 ± 0.15	2.35 ± 0.16	−0.22 ± 0.15
(N = 25) Contr SAS Item 7	2.28 ± 0.14	2.28 ± 1.07	0.0 ± 0.22
I am bothered by headaches and neck and back pain.			F = 22.669, *p* < 0.0001

**Table 3:** Differences in modifications in SAS Global Score and each SAS item score by time, comparing between groups—Part 02.

Experimental Group Control Group	T0	T1	Differences ANOVA 1-way 1; 63 df
(N = 39) Exper SAS Item 8	2.80 ± 1.02	2.48 ± 1.12	−0.32 ± 0.13
(N = 25) Contr SAS Item 8	2.40 ± 0.97	2.44 ± 1.02	+0.040 ± 0.18
I feel weak and get tired easily.			F = 52.156, *p* < 0.0001
(N = 39) Exper SAS Item 9	2.79 ± 0.99	2.66 ± 1.04	−0.13 ± 0.15
(N = 25) Contr SAS Item 9	2.96 ± 1.03	2.80 ± 1.05	−0.16 ± 0.18
I feel calm and can sit still easily.			F = 0.521, *p* = 0.437
(N = 39) Exper SAS Item 10	1.94 ± 0.95	1.79 ± 0.95	−0.15 ± 0.14
(N = 25) Contr SAS Item 10	2.04 ± 0.99	1.76 ± 0.86	−0.28 ± 0.18
I can feel my heart beating fast.			F = 10.485, *p* = 0.002
(N = 39) Exper SAS Item 11	2.17 ± 1.21	2.17 ± 1.10	−0.0 ± 0.15
(N = 25) Contr SAS Item 11	2.12 ± 1.17	2.04 ± 1.03	−0.8 ± 0.21
I am bothered by dizzy spells.			F = 2.759, *p* = 0.102
(N = 39) Exper SAS Item 12	1.74 ± 0.92	1.51 ± 0.87	−0.23 ± 0.13
(N = 25) Contr SAS Item 12	1.68 ± 1.00	1.56 ± 0.80	−0.12 ± 0.16
I have fainting spells or feel like it.			F = 9.095, *p* = 0.004
(N = 39) Exper SAS Item 13	2.23 ± 1.14	1.94 ± 1.13	−0.29 ± 0.23
(N = 25) Contr SAS Item 13	2.04 ± 1.03	2.20 ± 1.23	+16 ± 0.25
I can breathe in and out easily.			F = 22.630, *p* < 0.0001
(N = 39) Exper SAS Item 14	2.12 ± 1.09	1.97 ± 1.09	−0.15 ± 0.12
(N = 25) Contr SAS Item 14	2.00 ± 1.05	1.84 ± 1.04	−0.16 ± 0.15
I get numbness and tingling in my fingers and toes.			F = 0.08, *p* = 0.769
(N = 39) Exper SAS Item 15	2.05 ± 1.06	1.82 ± 1.00	−0.23 ± 0.11
(N = 25) Contr SAS Item 15	1.96 ± 1.14	2.00 ± 1.13	+0.04 ± 0.22
I am bothered by stomach aches or indigestion.			F = 39.816, *p* < 0.0001

**Table 4:** Differences in modifications in SAS Global Score and each SAS item score by time, comparing between groups—Part 03.

Experimental Group Control Group	T0	T1	Differences ANOVA 1-way 1; 63 df
(N = 39) Exper SAS Item 16	2.43 ± 1.10	2.28 ± 1.13	−0.15 ± 0.18
(N = 25) Contr SAS Item 16	2.36 ± 1.01	2.36 ± 1.12	0.00 ± 0.19
I have to empty my bladder often.			F = 10.132, *p* = 0.002
(N = 39) Exper SAS Item 17	2.51 ± 1.10	2.23 ± 1.16	−0.28 ± 0.21
(N = 25) Contr SAS Item 17	2.44 ± 1.23	2.56 ± 1.13	+0.12 ± 0.23
My hands are usually dry and warm.			F = 51.309, *p* < 0.0001
(N = 39) Exper SAS Item 18	1.84 ± 1.00	1.79 ± 0.97	−0.05 ± 0.17
(N = 25) Contr SAS Item 18	2.00 ± 1.01	1.64 ± 0.84	−0.36 ± 0.24
My face gets hot and blushes.			F = 36.592, *p* < 0.0001
(N = 39) Exper SAS Item 19	2.87 ± 1.20	2.79 ± 1.18	−0.08 ± 0.16
(N = 25) Contr SAS Item 19	3.16 ± 1.08	2.84 ± 1.11	−0.32 ± 0.23
I fall asleep easily and get a good night’s rest.			F = 24.262, *p* < 0.0001
(N = 39) Exper SAS Item 20	2.15 ± 0.97	2.02 ± 1.04	−0.13 ± 0.11
(N = 25) Contr SAS Item 20	2.12 ± 1.14	1.88 ± 0.99	−0.24 ± 0.24
I have nightmares			F = 6.201, *p* = 0.015

**Table 5:** Differences in modifications in SAS Global Score by time, comparing between sub-groups with or without non-psychiatric illness in comorbidity.

SAS in Participants with BD with Chronic Non-Psychiatric Illness			
	T0	T1	Differences
(N = 19) Exper	62.57 ± 17.40	60.05 ± 16.50	−2.53 ± 4.66
(N = 10) Contr	64.80 ± 14.62	61.00 ± 17.14	−3.80 ± 5.90
			F = 0.917, *p* = 0.342
SAS in Participants with BD without Non-Psychiatric Illness			
	T0	T1	Differences
(N = 20) Exper	59.60 ± 17.05	47.55 ± 18.7	−12.05 ± 5.24
(N = 15) Contr	55.10 ± 16.40	55.93 ± 16.74	0.83 ± 5.7
			F = 71.101, *p* < 0.0001

**Table 6:** Participants under the cut-off at SAS score for anxiety disorder by time and group.

	T0	T1	Gain	Fisher
Experimental Group	8 [20.5%]	15 [38.5%]	17.9%	*p* = 0.025
Control Group	6 [24%]	6 [24%]	0	
Participants with BD with chronic illness under the cut-off at SAS score for anxiety disorder				
	T0	T1	Gain	Fisher
Experimental Group	4 [21%]	4 [21%]	0	*p* = 0.111
Control Group	0	2 [20%]	20%	
Participants with BD without chronic illness under the cut-off at SAS score for anxiety disorder				
	T0	T1	Gain	Fisher
Experimental Group	4 [21.1%]	9 [47.3%]	5 [23.3%]	*p* = 0.042
Control Group	6 [60%]	4 [40%]	0	

## Data Availability

The data presented in this study are available upon request from the corresponding author. The data are not publicly available due to privacy and ethical issues.
